# Bovine semen sexing: Sperm membrane proteomics as candidates for immunological selection of X‐ and Y‐chromosome‐bearing sperm

**DOI:** 10.1002/vms3.540

**Published:** 2021-05-26

**Authors:** Joana Quelhas, Joana Santiago, Bárbara Matos, António Rocha, Graça Lopes, Margarida Fardilha

**Affiliations:** ^1^ Bovine Semen Collection and Storage Centre of Lusogenes Aveiro Portugal; ^2^ Laboratory of Signal Transduction Department of Medical Sciences Institute of Biomedicine – iBiMED University of Aveiro Aveiro Portugal; ^3^ Department of Veterinary Clinics Institute of Biomedical Sciences Abel Salazar‐University of Porto Porto Portugal; ^4^ Department of Imuno‐Physiology and Pharmacology Institute of Biomedical Sciences Abel Salazar‐ University of Porto Porto Portugal

**Keywords:** bovine, plasma membrane, proteomics, sexed semen, sperm

## Abstract

The use of sexed semen in dairy and beef farms ensures the production of animals of the desired sex, resulting in a reduction of costs and an improvement of environmental sustainability. Several methods have been developed over the years, but most of them were abandoned due to their limited efficacy. Currently, the only commercially available method for the separation of X‐ and Y‐chromosome‐bearing sperm is fluorescence‐activated cell sorting. However, this technique is expensive and has limited usefulness for the industry, considering that it cannot produce doses of sexed semen with the desired number of sperm for artificial insemination. Immunological methods have emerged as an attractive alternative to flow cytometry and proteomic knowledge of X‐ and Y‐sperm could be useful to the development of a new method. In this review, we identify the main applications of sexed semen, describe the existing methods and highlight future research opportunities in the field. We consider that immunological methods, based on sperm cell's surface proteins differentially expressed between X‐ and Y‐sperm, could be an interesting and promising approach to semen sexing.

## Graphical abstract

Immunological methods have emerged as an attractive alternative to flow cytometry and proteomic knowledge of X‐ and Y‐sperm could be useful to the development of a new method. In this review we identify the main applications of sexed semen, describe the existing methods and highlight the future research opportunities in the field. We consider that immunological methods, based on sperm cell's surface proteins, deferentially expressed between X‐ and Y‐sperm could be an interesting and promising approach to sex selection.

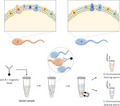

## 1 INTRODUCTION

Sexed semen is characterized by the presence of either X‐ or Y‐chromosome‐bearing sperm, allowing the production of offspring of the desired sex. Manipulating the sex of the animals has become of great interest to the industry, due to several sex‐related traits, like milking, herd replacement and growth rates. The use of sexed semen emerged in the 1980s after the development of artificial insemination (AI) and semen freezing techniques, and became a major resource in farms in the early 1990s (Garner & Seidel, 2008; Moore & Hasler, 2017). Despite its advantages, the sperm concentration of sexed semen is far less than the conventional semen straw and the sorting procedure usually causes physical/physiological damage to the sperm, compromising fertilization results (Grant & Chamley, 2007).

In the last decades, several unsuccessful studies and many inoperative patents for the sex separation of sperm emerged. Currently, there is only one quantitative and reasonably accurate method for sexing mammalian sperm available, that consists of individual discrimination and separation of X‐ and Y‐chromosome‐bearing sperm through flow cytometry sex‐sorting (Garner & Seidel, 2008). Considering the limitations of the current technologies, the future of sexing technologies seems to have many perspectives (Moore & Hasler, 2017). Finding an improved methodology for sperm selection that increases the fertilization ability of sex‐separated sperm is the ultimate goal (Moore & Hasler, 2017).

The knowledge and comparison of the protein repertoire of X‐ and Y‐sperm can be useful to develop alternative separation methods, making the application of proteomic methodologies promising in the future of sexing semen technologies (De Canio et al., 2014). This review addresses the main applications of sexed semen, summarizes the principal methods developed in the past decades and highlights future research opportunities in the field, focusing on the importance of the development of a new immunological approach for semen sexing based on X‐ and Y‐sperm‐specific plasma membrane proteins.

## 2 APPLICATIONS OF SEXED SEMEN

There is an increasing demand for dairy and beef products worldwide, which requires a great focus on improving production efficiency. The use of sexed semen in dairy and beef cattle production provides several benefits at both farm and industry levels. In particular, the use of this technology can increase the efficiency of both dairy and beef production, increase farm profitability and improve the environmental sustainability of cattle agriculture (Holden & Butler, 2018). In dairy farming, semen sorting may overcome the surplus production of unwanted male calves which, as an unwanted byproduct of breeding with conventional semen, have low economic value (Holden & Butler, 2018). In the beef‐cattle industry there is an urgent need to improve the production yield, which can be done by raising more heifers from high‐quality cows, resulting in superior replacement of females and donors (Hall & Glaze, 2014). Ultimately, the availability of sexed semen would allow the selection of the best bulls and cows (Holden & Butler, 2018).

However, the decreased fertilization potential and impairment of embryonic development of sex‐sorted semen, when compared with conventional semen, are consequences of: (a) the reduction in sperm concentration and motility in most commercial doses and (b) the damage caused during sorting procedures (Mikkola & Taponen, 2017; Seidel, 2014). Sex‐sorted sperm produced also have reduced viability and overall quality after cryopreservation and thawing (Seidel & Garner, 2002). The reduced pregnancy rates obtained with these doses result in indirect costs for the livestock industry, which suggest that optimization of the currently used methods is needed (Garner & Seidel, 2008; Moore & Hasler, 2017; Rath & Johnson, 2008).

## 3 CURRENTLY AVAILABLE SEMEN SEXING METHODS

Several features allow distinguishing X‐ and Y‐sperm: (a) the DNA content (X‐sperm contains more DNA than Y‐sperm); (b) the size (X‐sperm is larger than Y‐sperm); (c) the charge on the cell's surface (X‐sperm has a negative charge and Y‐sperm has a positive charge) and (iv) the motility pattern (X‐sperm has lower motility than Y‐sperm). The surface antigens are also different between X‐ and Y‐sperm (Cui, 1997; Garner & Seidel, 2008; Yadav et al., 2017). Based on these distinguishing features, several sexing methods have been developed. In the following sections, the most popular methods developed to separate X‐ and Y‐sperm and their main advantages and limitations are described.

### 3.1 Sexing techniques based on kinetics and physical properties of X‐ and Y‐sperm

The first methods used to enrich semen in X‐ or Y‐sperm were based on their potential differences in the kinetics and physical properties (amount of DNA, size, density and motility of the sperm cell) (Ericsson et al., 1973; Kaiser et al., 1974; Soupart & Strong, 1975; Steeno et al., 1975; Han et al., 1993). These techniques assume that Y‐sperm has less DNA, is smaller, has higher motility and less density than X‐sperm. In particular, the Percoll^®^ gradient centrifugation technique allows the separation of X‐ and Y‐sperm based on the difference in their density (Kaneko et al., 1983). The albumin gradient method is based on the differences in sperm motility in bovine serum albumin solutions of various concentrations (Ericsson et al., 1973). The differences in swimming speed between X‐ and Y‐sperm were also used for sperm sex sorting through a method called swim‐up (Han et al., 1993). Finally, Sephadex columns restricted the diffusion of the cells by a certain porosity, allowing the separation of X‐ and Y‐sperm based on their size differences (Steeno et al., 1975).

Initially, the simplicity of these technologies and the equipment employed, which were associated with low costs, were attractive to many scientists. However, these techniques missed satisfactory results to be reproduced and applied in the commercial market, due to the variability of the results and the poor efficiency in the separation of sperm (Barros Mothé et al., 2018; Ellis et al., 2011; Koundouros & Verma, 2012). Several attempts to optimize these methods were developed, with a maximal accuracy of approximately 65%–75%, which was unsatisfactory and triggered the development of new sexing methods (Resende et al., 2011).

### 3.2 Flow cytometry sex‐sorting

Currently, there is only one method used for commercial sex‐sorting, which consists in the individual separation of X‐ and Y‐chromosome‐bearing sperm using flow cytometry—fluorescence‐activated cell sorting (FACS) (Garner et al., 2013; Moore & Hasler, 2017; Seidel, 2014). This technology was patented in 1991 by the United States Department of Agriculture and related technologies have been licensed and sub‐licensed over the years (Johnson, 2000). In this method, sperm cells are exposed to a fluorescent dye (Hoechst 33342) that binds to intact DNA and are analysed by flow cytometry (Ellis et al., 2011; Moore & Hasler, 2017; Rath & Johnson, 2008; Seidel, 2014). Since bovine X‐bearing sperm contains about 4% more DNA than Y‐sperm, it emits a brighter fluorescence, allowing the differentiation of the two subpopulations (Ellis et al., 2011; Moore & Hasler, 2017; Rath & Johnson, 2008; Seidel, 2014). The fluorescently stained sperm are sorted using a specialized high‐speed sorter, and collected into the biologically supportive medium before cryopreservation in adequate doses to be used for AI or in vitro fertilization (IVF) (Moore & Hasler, 2017; Rath & Johnson, 2008; Vishwanath & Moreno, 2018).

Offspring of several species (sheep, bovine, rabbit, pig, horse, dog, cat and dolphin) was produced with this method (Garner, 2006), but only bovine sexed semen can be found commercially, encouraged by demand for females in dairy farming (Garner, 2006). The application of this method in both dairy and beef production allows 75%–90% accuracy in sex selection, which is a great advantage when compared with other methods (Garner and Seidel 2000; Holden & Butler, 2018; Moore & Hasler, 2017; Seidel, 2014). However, compared with conventional semen, this method has higher costs and time of production (only 7–12 straws per hour), and a lower number of sperm per straw (2–4 million; Moore & Hasler, 2017; Rath et al., 2015). Also, sperm may suffer damage during the procedure that might compromise their motility and viability affecting the fertilization potential and embryonic development (Garner and Seidel 2000; Rath et al., 2015; Seidel, 2014; Thomas et al., 2019). The success of this technique depends on the immediate semen sorting after collection (less than 7 hr), requiring the adaptation of the laboratory dynamics (Kaiser et al., 1974; Seidel, 2014). Improved semen quality and conception rates were achieved with the recent SexedULTRA^TM^ technology (Brito et al., 2019). Thus, despite the substantial improvements of this technology throughout the years, a more efficient and economic method for the separation of X‐ and Y‐sperm with a minimal impact on sperm morphology and physiology, and with a high success rate should be developed.

## 4 IMMUNOLOGICAL METHODS FOR SEMEN SEXING

The use of FACS for sex‐sorting provided new opportunities to the study of X‐ and Y‐sperm populations, allowing the investigation of molecular properties that can be used to design new methods of sex selection. Immunological methods have emerged for sexing sperm, based on differences in protein expression of X‐ and Y‐sperm (Katigbak et al., 2019; Yadav et al., 2017). This promising technology is based on the assumption that the genomic differences among X‐ and Y‐sperm might result in protein and functional differences as well, and that the gene product is confined to the sperm carrying that chromosome (Howes et al., 1997). Recently, a sperm‐sexing method based on the activation of Toll‐like receptor 7/8 (TLR7/8) on X‐sperm was developed (Umehara et al., 2019, 2020). TLR7 and TLR8 are localized inside cells and were detected only in X‐sperm tail and midpiece, respectively (Umehara et al., 2019). Umehara and colleagues used the TLR7/8 ligand resiquimod (R848) to activate TLR7/8 in mouse and bull X‐sperm, resulting in decreased glycolytic activity and ATP production, with a consequent reduction in X‐sperm motility (Umehara et al., 2019). With this approach, X‐sperm remained in the lower layer and most of the upper layer contained highly motile Y‐sperm (Umehara et al., 2020). In bulls, the use of the upper layer for IVF resulted in 91.3 ± 2.8% of XY embryos. The motility of the lower layer can be rescued by removing R848 from the medium, allowing the production of XX embryos (84.2 ± 5.3%) using this fraction (Umehara et al., 2020). However, this method needs further optimization to be applied to freshly ejaculated sperm and to be used for AI.

There is evidence that surface‐specific antigens found in X‐ and Y‐sperm can potentially be used to separate them (Sang et al., 2011; Yadav et al., 2017). This idea arises from the observation that HY antigen, which is exclusively found in mammalian tissues, could be used as an immunological marker (Wachtel et al., 1975). Several researchers showed a correlation between anti‐HY antibody binding and the presence of Y‐chromosome‐bearing sperm. However, there are some conflicting results in the literature about the difference in expression of this antigen in X‐ and Y‐sperm, and there are even studies that confirmed the presence of this antigen in both (Bradley, 1989; Hendriksen et al., 1993). Thus, a sexing method based on the anti‐HY antibody becomes inappropriate and cannot be used to differentiate between X‐ and Y‐spermatozoa.

The search for the identification of specific proteins in X‐ and Y‐sperm has required several improved techniques, especially proteomics which has been considered a crucial tool in several studies involving sperm (Katigbak et al., 2019; Yadav et al., 2017). It allowed the identification of protein snapshots in different infertility‐related conditions, as well as to differentiate X‐ and Y‐sperm (De Canio et al., 2014; Yadav et al., 2017). Currently, few studies exist regarding bovine sperm proteome (Kasvandik et al., 2015; Soggiu et al., 2013) and only a few investigated the differential protein profile in sexed bovine semen (Table 1) (Chen et al., 2012; De Canio et al., 2014; Howes et al., 1997; Scott et al., 2018). The first study that addressed the identification of differentially expressed surface proteins between populations of X‐ and Y‐sperm was done by Howes and colleagues and failed to identify any difference (Howes et al., 1997). Later, an extensive proteomic investigation of sexed sperm cells by two‐dimensional gel electrophoresis (2‐DE)/MS revealed differentially expressed proteins between bull X‐ and Y‐ sperm, that included proteins involved in energy metabolism, cytoskeleton and inhibitors of serine proteases (Chen et al., 2012) (Table 1). De Canio et al. (2014) also developed a comparative study by nano ultra‐performed liquid chromatography‐tandem mass spectrometry (nUPLC‐NS/MS), to characterize bovine sexed semen samples. In this study, 17 unique proteins were found: 15 exclusively present in X‐sperm and 2 in Y‐sperm (Table 1). These proteins were associated with the structural cytoskeleton of the flagellum, glycolytic enzymes and calmodulins (De Canio et al., 2014). Recently, Scott et al. (2018) used a SWATH‐MS analysis to profile proteins of sperm previously separated by flow cytometry into X‐ or Y‐bearing semen pools. The authors recognized eight proteins differentially expressed between the X‐ and Y‐bearing sperm populations (Table 1). Furthermore, these proteomic analyses of X‐ and Y‐sperm in bulls revealed that F‐actin capping protein subunit beta 2 (CAPZB) and Cytochrome b‐c1 complex subunit, mitochondrial (UQCRC1) have different expression levels and these differences seem to affect the phenotype of X‐ and Y‐sperm. Altogether, around 40 proteins differentially expressed in bovine X‐ and Y‐sperm that were related to the energetic metabolism, structural cytoskeleton, stress resistance and protein serine activity were identified (Chen et al., 2012; De Canio et al., 2014; Yadav et al., 2017). These studies highlight that these proteins may be used to understand the difference between the two types of sperm and may contribute to the development of immune‐sexing techniques.

**TABLE 1:** Differentially expressed proteins between X‐ and Y‐bovine sperm. Information regarding studies, Uniprot ID, gene name and protein name were included

References	Uniprot ID	Gene name	Protein name
*Proteins upregulated in X‐bearing bovine sperm*			
Scott et al. (2018)	Q8MJN0	FUNDC2	FUN14 domain‐containing protein 2
	F1MSC3	ACACB	Acetyl‐CoA carboxylase, type beta
	P42026	NDUFS7	NADH dehydrogenase [ubiquinone] iron‐sulphur protein 7, mitochondrial
De Canio et al. (2014)	P02784		Seminal plasma protein PDC 109
	P10096	GAPDH	Glyceraldehyde 3 phosphate dehydrogenase
	Q2T9U2	ODF2	Outer dense fiber protein 2
	Q3ZBU7	TUBB4A	Tubulin beta 4ª
	P19858	LDHA	L‐lactate dehydrogenase A
	Q29438	ODF1	Outer dense fibre protein 1
	O77797	AKAP3	A kinase anchor protein 3
	Q32LE5	ASRGL1	L‐asparaginase
	Q3MHM5	TUBB4B	Tubulin beta 4B
	Q32KN8	TUBA3	Tubulin alpha 3
	Q2TBH0	ODF3	Outer dense fibre protein 3
	Q2KJE5	GAPDHS	Glyceraldehyde 3 phosphate dehydrogenase testis specific
	Q2YDG7	SPACA1	Sperm acrosome membrane associated protein 1
	Q5E956	TPI1	Triosephosphate isomerase
	P62157	CALM	Calmodulin
Chen et al. (2012)	F1MWY0	NSMAF	Similar to neutral sphingomyelinase (*N*‐SMase) activation associated factor
			oxidase heme a, cytochrome
	P31800	UQCRC1	Cytochrome b–c1 complex subunit 1, mitochondrial
	AAI05544	HIBADH	3‐hydroxyisobutyrate dehydrogenase
	Q32KN8	TUBA3	Tubulin alpha‐3 chain
	P41563	IDH3A	Isocitrate dehydrogenase [NAD] subunit alpha, mitochondrial
			Chain A, the structure of crystalline profilin‐beta‐actin
			A Chain A, episelection: Novel Ki~nanomolar inhibitors of serine proteases selected by binding or chemistry on an enzyme surface
	Q3MHM5	TUBB4B	Tubulin beta 4B
*Proteins upregulated in Y‐bearing bovine sperm*			
Scott et al. (2018)	E1BKH1	EFHC1	EF‐hand domain‐containing protein 1
	E1BPM9	DNAI2	Dynein intermediate chain 2, axonemal
	P22439	PDHX	Pyruvate dehydrogenase protein X componente
	Q2HJ55	SAMM50	Sorting and assembly machinery component 50 homolog
	P68530	COX2	Cytochrome c oxidase subunit 2
De Canio et al. (2014)	Q2HJB8	TUBA8	Tubulin alpha 8
	Q6B856	TUBB2B	Tubulin beta 2B
Chen et al. (2012)			Chain A, crystal structure of bovine heart mitochondrial Bc1 with Jg144 inhibitor
	P00829	ATP5F1B	ATP synthase subunit beta, mitochondrial
	P79136	CAPZB	F‐actin‐capping protein subunit beta
		GSTM3	Glutathione‐S‐transferase, mu 3 (brain)

Immunological methods seem to be promising to separate X‐ and Y‐sperm and appear to be less aggressive to sperm and economically more viable than the currently available methods (Yadav et al., 2017). However, these methods must be carefully studied and developed for consistent results (Yadav et al., 2017). X‐ or Y‐sperm proteins located on sperm membrane surface represent the best candidates for immunological selection. If one protein can be exclusively identified on X‐ or Y‐sperm surface, then antibodies could be developed against it. This is particularly important since proteins located inside spermatozoa are not recognized by antibodies unless a permeabilization step is included in the procedure, compromising the viability of those cells and their use for fertilization procedures. Subsequently, the use of magnetic beads, affinity chromatography or FACS can be adopted to provide a batch separation. Recently, the use of antibodies against the surface antigen Sex‐determining Region Y (SRY), localized specifically in bovine Y‐sperm, for semen sexing has been described (Soleymani et al., 2019, 2020). According to Soleymani et al. (2019), the polyclonal goat anti‐rbSRY antibody developed is capable of specifically binding to Y‐chromosome‐bearing spermatozoa while it does not bind X‐chromosome‐bearing spermatozoa. Also, the same authors recently produced a monoclonal antibody against rbSRY (SRY2mab), that was bound to Sepharose columns (Soleymani et al., 2020). Only Y‐sperm bound to the columns, suggesting that this new approach can be potentially used for the proper separation of X‐ and Y‐sperm (Soleymani et al., 2020). However, this separation protocol significantly reduced sperm motility (Soleymani et al., 2020). Chowdhury and colleagues also described the use of a monoclonal antibody that binds specifically to the plasma membrane of the Y‐sperm heads—WholeMom^®^—for the separation of X‐ and Y‐sperm and the production of cattle embryos with pre‐selected sexes (Chowdhury et al., 2019). Developed against bull Y‐sperm epitopes, WholeMom^®^ does not bind to the X‐sperm and the results showed that no differences were observed in cleavage and blastocyst developmental rates between the use of X‐sperm selected by this method and conventional semen (Chowdhury et al., 2019). However, this approach was applied to cryopreserved samples and should be reproduced using fresh semen. Despite this evidence, no efficient method using this technology has been presented at this time.

## 5 DIFFERENCES IN X‐ AND Y‐SPERM PLASMA MEMBRANE PROTEINS OPEN THE DOORS TO THE DEVELOPMENT OF NEW IMMUNOLOGICAL SEMEN SEXING APPROACHES

**FIGURE 1:**
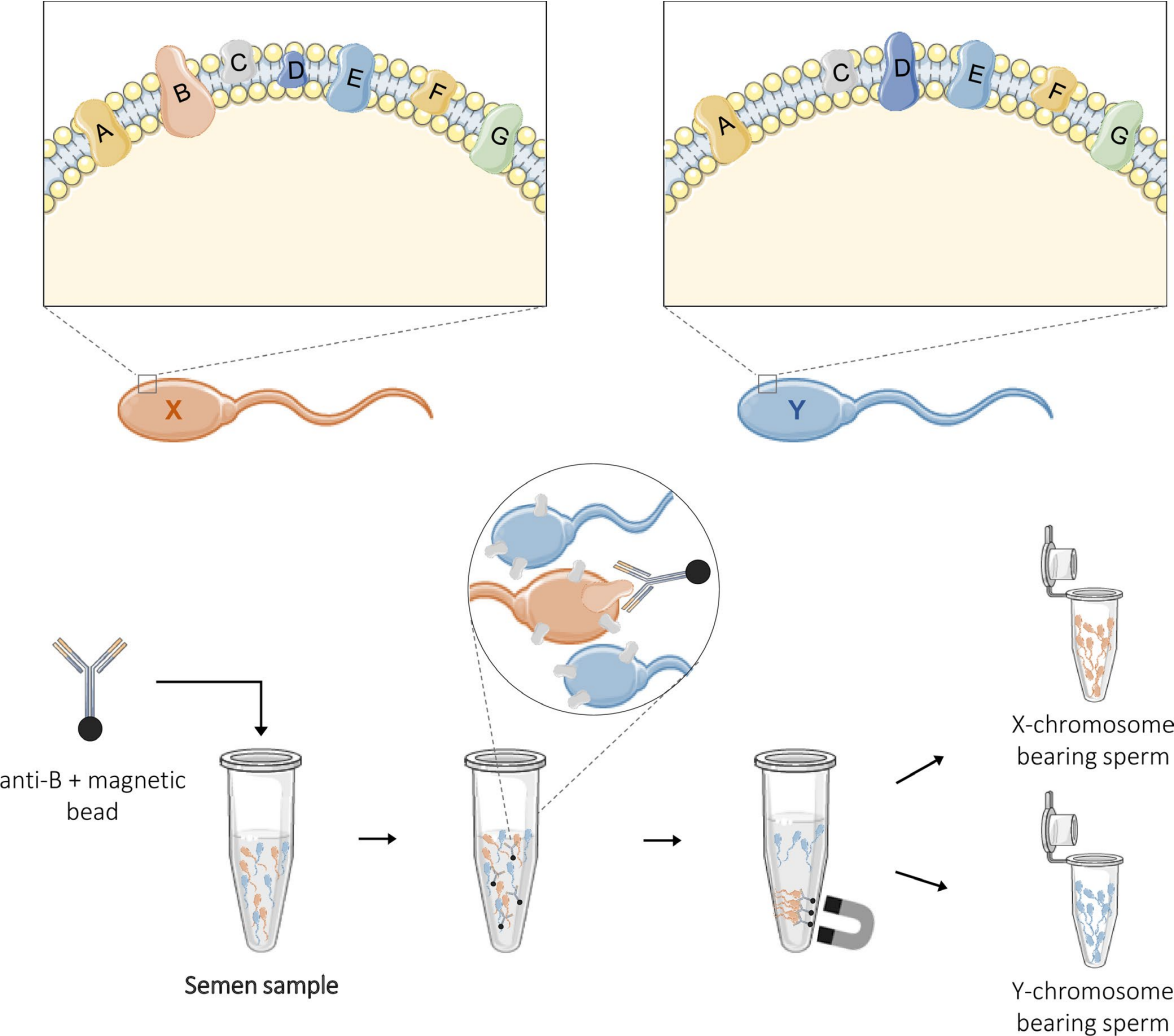
Method proposed for the separation of X‐ and Y‐chromosome‐bearing sperm based on differences on plasma membrane protein content. For example, plasma membrane of X‐sperm contains protein A, B, C, D, E, F and G, but in Y‐sperm plasma membrane, protein B was absent. The development of an anti‐B antibody, against protein B exclusively expressed in X‐sperm, will allow the recognition of X‐sperm only. This anti‐B antibody can be coupled to magnetic beads to immunocapture the desired cell population (X‐sperm) allowing its separation from Y‐sperm, with less damage and potentially less sperm loss than the currently available methods

As already mentioned, immunological methods seem to be promising to separate X‐ and Y‐sperm, being crucial the identification of uniquely expressed proteins in X‐ and Y‐ sperm. Sperm surface components of the two populations are of particular interest to develop new immunological methods. Indeed, differences in plasma membrane antigenicity may be explored to generate specific antibodies able to separate X‐ and Y‐chromosome‐bearing sperm, without affect sperm integrity. For example, specific antibodies can be coupled to magnetic beads to immunocapture the desired cell subpopulation (X‐ or Y‐sperm) or methods to recover the sperm presenting the target candidates in antibody‐coated dishes can also be adopted (Figure 1). Thus, it is important to identify differential expressed plasma membrane proteins in bovine X‐ and Y‐sperm that have the potential to integrate a cost‐effective and non‐invasive method of sperm sex selection, possible to be used immediately after the semen collection or immediately before the AI to avoid several cryopreservation cycles.

To identify possible plasma membrane proteins exclusively, or at least differentially, expressed in X‐ and Y‐sperm, an extensive literature search was conducted in the PubMed database using the keywords “bovine”, “semen” and “proteome” or “proteomics”, to identify bovine sperm plasma membrane proteomic studies and to retrieve differentially expressed proteins in bovine X‐sperm and Y‐sperm. A list of all the proteins identified in the proteomic studies available online until 26 February 2019 was compiled. Only studies using ejaculated bovine sperm, published in English and only proteins identified with at least two peptides were included. All proteins were annotated using the UniProtKB/Swiss‐Prot accession number. To obtain the list of plasma membrane proteins differentially expressed in X‐ and Y‐sperm, Venn diagram analysis was performed using the Jvenn tool (Bardou et al., 2014). By merging data from the available papers and after removing the duplicates, a list of 456 proteins present in the bovine sperm plasma membrane was created, which we considered that are very likely to compose the bovine sperm plasma membrane proteome. Additionally, 26 proteins upregulated in X‐sperm and 11 proteins upregulated in Y‐sperm were identified (Table 1).

Gathering the bovine sperm plasma membrane proteome and crossing it with the differentially expressed proteins identified in X‐ and Y‐bovine sperm, we identified 12 plasma membrane proteins upregulated in bovine X‐sperm and three upregulated in bovine Y‐sperm (Table 2). From those, the sperm acrosome membrane associated protein 1 (SPACA1), upregulated in X‐chromosome‐bearing sperm, constitute the best candidate for an antibody‐based separation of sexed sperm cells. This 34 kDa membrane protein is abundant in bovine semen and is primarily found in the equatorial segment of the acrosome, which provides a great opportunity for selecting spermatozoa while they are alive. In fact, as already mentioned, the use of plasma membrane proteins avoids the need for sperm permeabilization, necessary for antibodies to bind to the proteins intracellularly. Given the relevance of this question for the development of alternative immunological methods of sorting, it should be investigated by a direct experimental approach for the selective targeting of sperm surface proteins through antibodies or a selective technique of labelling. Additionally, the use of sperm‐specific proteins such as SPACA1, allows the selection of only spermatozoa, avoiding contamination by other somatic cells with the same antigen on the surface. Of note, some of the candidates presented in Table 2 are annotated as cytosolic and/or cytoskeleton proteins for instance, thus, the antibodies accessibility of these proteins is not obvious and needs further validation. Avoiding contamination by subcellular organelles and cytosolic proteins is crucial to the successful proteomic analysis of integral plasma membrane proteins.

**TABLE 2:** Plasma membrane (PM) proteins differentially expressed in X‐ and Y‐bovine sperm along with their Uniprot ID and gene name

Uniprot ID	Gene name	Protein name
*Common elements in PM bovine X‐sperm:*		
O77797	AKAP3	A kinase anchor protein 3
P02784		Seminal plasma protein PDC 109
P19858	LDHA	L‐lactate dehydrogenase A
P62157	CALM	Calmodulin
Q2KJE5	GAPDHS	Glyceraldehyde 3 phosphate dehydrogenase testis specific
Q2T9U2	ODF2	Outer dense fibre protein 2
Q2YDG7	SPACA1	Sperm acrosome membrane associated protein 1
Q32KN8	TUBA3	Tubulin alpha 3
Q32LE5	ASRGL1	L‐asparaginase
Q3MHM5	TUBB4B	Tubulin beta−4B chain
Q3ZBU7	TUBB4A	Tubulin beta 4ª
Q5E956	TPI1	Triosephosphate isomerase
*Common elements in PM bovine Y‐sperm:*		
P00829	ATP5F1B	ATP synthase subunit beta, mitochondrial
P79136	CAPZB	F‐actin‐capping protein subunit beta
Q6B856	TUBB2B	Tubulin beta 2B

We consider that investing in this approach, and in the characterization of bovine sperm plasma membrane proteins differentially expressed in X‐ and Y‐sperm will be of great interest not only for industry but also for basic researchers. Indeed, it will allow the recognition of plasma membrane‐specific markers of sperm carrying X‐ and Y‐chromosome that have the potential to integrate a cheaper, efficient and scalable method of sex selection, easier to use in the daily routine of livestock industries. Additionally, this approach has great value for basic research, allowing the study of X‐ and Y‐sperm separately and providing a base for a better comprehension of the biological features of sperm cells.

## 6 CONCLUSION

The use of sexed semen is a pointed way to optimize meat and milk production. The development and study of alternative techniques and the improvement of the current ones have been a constant in this field; however, the currently available commercial method still presents limitations (high cost, low sperm number per straw and sperm damage).

The sperm membrane is a logical target for the development of a new sperm selection method. The use of knowledge in X‐ and Y‐sperm plasma membrane protein content is a useful approach to develop an efficient, non‐invasive and low‐cost method of sexing sperm. The identification of plasma membrane proteins uniquely expressed in X‐ and Y‐sperm will enable the generation of specific antibodies that will recognize these unique antigens, allowing their separation with less damage and sperm loss. This approach has the potential to increase the inseminating dose, reduce the costs of the procedure and be more efficient in the separation. Ultimately, the development of this new method will improve the efficiency of both dairy and beef production, increase farm profitability and enhance the environmental sustainability of cattle agriculture.

## AUTHOR CONTRIBUTIONS

Joana Quelhas: Conceptualization; Investigation; Methodology; Writing‐original draft. Joana Santiago: Conceptualization; Data curation; Formal analysis; Investigation; Validation; Writing‐original draft; Writing‐review & editing. Bárbara Matos: Investigation; Writing‐original draft; Writing‐review & editing. Antonio Rocha: Funding acquisition; Supervision; Writing‐review & editing. Graça Lopes: Funding acquisition; Supervision; Writing‐review & editing. Margarida Fardilha: Conceptualization; Funding acquisition; Project administration; Supervision; Writing‐review & editing.

## Data Availability

Data sharing is not applicable to this article as no new data were created or analysed in this study.
